# Clinical Innovation Poster Abstracts

**DOI:** 10.1080/24740527.2018.1476311

**Published:** 2018-05-21

**Background and Aims**: Over the past two-years ACC has completed a redesign of its pain management services to address key delivery challenges. An action research approach was utilized to support the co-design of these services to ensure the redesigned service best met the needs of patients, clinicians and ACC staff. A new service delivery model was developed and refined in conjunction with eight pain management services in Auckland, New Zealand.

**Methods**: An independent research organization was engaged to undertake action research to support the development of the service design. Completed in three phases, this included:

1. An evaluation design and context phase
2. Data collection and formative feedback
3. Mixed methods data integration and analysis

Information was gathered over a period of three months with rapid feedback provided to ACC every two-weeks.

**Conclusions**: The use of an action research approach to service co-design was invaluable in developing a new model of pain management in New Zealand. This approach allowed incremental changes to be made based on participant feedback and tested in the context in which they are delivered. This allowed for only those changes which improved client outcome and experience to be adopted in the new service. Key changes included triage, expectation setting, early intervention, cross agency collaboration and staff training. In addition, this approach increased engagement and satisfaction of clinicians working in the services. The new model has now been implemented nationally with outcomes benchmarked across Australasia using ePPOC (electronic Persistent Pain Outcomes Collaboration).

## The role of action research in service design – experience from the accident compensation corporation New Zealand

Kristin Good

**Background and Aims**: Over the past two-years ACC has completed a redesign of its pain management services to address key delivery challenges. An action research approach was utilized to support the co-design of these services to ensure the redesigned service best met the needs of patients, clinicians and ACC staff. A new service delivery model was developed and refined in conjunction with eight pain management services in Auckland, New Zealand.

**Methods**: An independent research organization was engaged to undertake action research to support the development of the service design. Completed in three phases, this included:

(1) An evaluation design and context phase
(2) Data collection and formative feedback
(3) Mixed methods data integration and analysis

Information was gathered over a period of three months with rapid feedback provided to ACC every two-weeks.

**Conclusions**: The use of an action research approach to service co-design was invaluable in developing a new model of pain management in New Zealand. This approach allowed incremental changes to be made based on participant feedback and tested in the context in which they are delivered. This allowed for only those changes which improved client outcome and experience to be adopted in the new service. Key changes included triage, expectation setting, early intervention, cross agency collaboration and staff training. In addition, this approach increased engagement and satisfaction of clinicians working in the services. The new model has now been implemented nationally with outcomes benchmarked across Australasia using ePPOC (electronic Persistent Pain Outcomes Collaboration).

**CONTACT** Kristin Good kristin.good2@acc.co.nz

## A new way to diagnose and treat low back pain

Hélène Bertrand

Clinical instructor, UBC Department of Family Practice, Vancouver, BC, Canada

**Introduction/Aim**: Low back pain, one of the commonest and costliest complaints is usually ascribed to lumbar spine pathology. A new way to examine the sacroiliac (SI) joint compares the alignment of the posterior superior iliac spines (PSIS).

**Methods**: 180 charts (2015 – 2017) were reviewed. If the sacro-tuberous ligament under a PSIS is tender, the corresponding SI joint is deemed affected. If the affected PSIS is higher than its mate, the SI joint is anteriorly subluxed. If it is lower it is posteriorly subluxed. Anterior subluxation is corrected by jamming the thigh on the affected side against the anterior superior iliac spine (ASIS) using a chair. Posterior subluxation correction involves hyperextending the thigh on the affected side. Each exercise is held for 2 minutes.

**Results**: The median duration of low back pain was 3 years. 16 cases had definite lumbar origin pain. In the other 164, using these maneuvers showed 96 had anterior subluxation, of whom 49 (51%) had complete and 30 (31%) had partial pain relief following the chair exercise. Of the 84 who had posterior subluxation, 43 (52%) had complete and 20 (24%) had partial pain relief after the thigh hyperextension exercise. 91 of 180 cases of low back pain (50%) found complete relief following these maneuvers, which they were taught to use for recurrences.

**Discussion/Conclusions**: 70% of low back pain patients found some relief from their pain using these SI joint corrective maneuvers, indicating the prevalence of this condition and a new way to treat it.

**CONTACT** Hélène Bertrand dr.hbertrand@gmail.com

### Integrating psychological, physical and pharmacological interventions for youth with chronic pain and their caregivers

Alina Carter, Lindsey Barrieau, Kim Prud'homme, and Julie Boucher

Children's Hospital of Eastern Ontario, Rehabilitation Program and Chronic Pain Service, Ottawa, Ontario, Canada

The Chronic Pain Service (CPS) at the Children's Hospital of Eastern Ontario (CHEO) is an interdisciplinary treatment team, which strives for an integrated evidence-based approach to chronic pain management for youth and their families. Due to high need and volume of patients, current services include serial waitlists for various disciplines involved in treatment (e.g., psychology, occupational therapy, physiotherapy). Based on themes identified through a needs assessment and a review of the literature on evidence-based treatments, CPS clinicians developed a group intervention that includes psychological, physical and pharmacological aspects of treatment. The program, which is based on existing evidence-based interventions, consists of eight, half-day sessions for youth and their caregivers.

The intervention was piloted early in 2017 among female chronic pain patients between the ages of 15 and 18 years ($n = 7$) and their caregivers. Youth and caregiver satisfaction surveys and pre and post evaluation measures were completed to help with program development and improvement. Perceived level of knowledge of pediatric chronic pain improved significantly in youth as a result of the intervention ($p < 0.05$) and caregivers reported learning new strategies to help their children manage their chronic pain ($p = 0.06$). The pilot study also helped identify which youth may benefit most from this intervention. Given the findings and feedback from participants and staff involved, this program is being revised, with the intent to develop it as an integral part of the pain service.

**CONTACT** Alina Carter dr.alina.carter@gmail.com Gilmour Psychological Services, 437 Gilmour Street, Ottawa, ON, K2P 0R5, Canada.

### Analysis of non-scheduled telephone calls received by a specialized cancer pain nurse. Descriptive analysis of calls and actions taken

Christelle Remy[a] and Jordi Perez[b]

[a]Nurse clinician, Cancer Pain Clinic, McGill University Health Centre, Montreal, Quebec, Canada; [b]Clinical director, Cancer Pain Clinic, McGill University Health Centre, Montreal, Quebec, Canada

**Introduction**: Nurses play a critical role in managing the patient's needs either in clinic or by telephone. Telephone consultations may help prevent symptoms from becoming unmanageable and possibly avoid unnecessary and costly visits to emergency departments. On the other side they may help identifying potential serious health problems requiring urgent medical care. We conducted a retrospective analysis of non-scheduled phone calls received at our cancer pain clinic with the objective of refining our services offered to patients by phone and finding a pattern of actions taken.

**Methods**: Retrospective chart review of phone calls attended by the cancer pain nurse for 3 months (Nov. 2016 to Feb. 2017). Data collected included caller identification, reason for the call and intervention(s) provided.

**Results**: 102 phone calls were received concerning 87 of our outpatients. Most calls were initiated by patients themselves. Main reasons for calling were symptom management (46%) and medication-related issues (45%). Pain was the most common symptom (60%) followed by side effects (23%). Of medication-related calls, drug renewal (48%) and request for further information (28%) were the 2 most commons. Most of actions needed concerned medications (74%) and included education about the therapy, drug renewal and adjustment of posology. Interestingly, 87% of interventions were provided without necessitating consulting a physician. 10% of cases were referred to another department and particularly, 5 patients were advised to present to ED.

**Conclusions**: The nurse clinician specialised in cancer pain plays a major role in providing continuity of care to outpatients seen in clinic. The easy access to phone consultation represents a major advantage to patients by reassuring their needs and avoiding unnecessary stress and suffering.

**CONTACT** Christelle Remy christelle.remy@muhc.mcgill.ca

© 2018 Christelle Remy and Jordi Perez. Published with license by Taylor & Francis Group, LLC.
This is an Open Access article distributed under the terms of the Creative Commons Attribution License (http://creativecommons.org/licenses/by/4.0/), which permits unrestricted use, distribution, and reproduction in any medium, provided the original work is properly cited.

### Targeting obesity in Australian chronic pain settings: A new model of care

Allyson Browne[a] and Stephan Schug[a,b]

[a]Anaesthesiology Unit, Medical School, University of Western Australia, Perth, Western Australia, Australia; [b]Pain Medicine Centre, Royal Perth Hospital, Perth, Western Australia, Australia

Obese and severely obese adults have been found to be twice and four times as likely respectively, compared to those of normal weight, to report chronic pain[1]. Obese patients have poorer treatment response, higher health care use, and up to 82% treatment drop-out rate compared to their non-obese counterparts[2,3] Yet, little attention has been given to the development of treatment programs that target *both* obesity and chronic pain. Our team is developing a new model of care that targets obesity as part of chronic pain management. Commencing in January 2018, all new patients attending the Pain Medicine Centre at Royal Perth Hospital will be assessed for weight-related factors (BMI, weight, height, waist-hip ratio, physical activity, food and beverage intake), in addition to pain-related factors (symptom and treatment history, medication use, pain catastrophizing, pain self-efficacy, pain-related disability), and specific psychosocial variables known to contribute to obesity and chronic pain symptoms (smoking and alcohol use, depression and anxiety symptoms, quality of life, emotional regulation strategy use, and trauma symptoms) using standardized measures. Findings from the first phase of the project will be presented identifying the prevalence of obesity among chronic pain patients, and modifiable psychological factors that predict obesity-related factors and pain outcomes. These measures will be repeated at 12 months to compare treatment response of obese and non-obese chronic pain patients, and guide the development of the second phase of the project targeting obesity specifically as part of the multidisciplinary approach to chronic pain management.

**CONTACT** Allyson Browne allyson.browne@uwa.edu.au

© 2018 Allyson Browne and Stephan Schug. Published with license by Taylor & Francis Group, LLC.
This is an Open Access article distributed under the terms of the Creative Commons Attribution License (http://creativecommons.org/licenses/by/4.0/), which permits unrestricted use, distribution, and reproduction in any medium, provided the original work is properly cited.

### Bridging the gap - The Sick Kids Transitional Pain Clinic: optimizing paediatric pain management from hospital to home/rehabilitation care

Lorraine Bird, Lisa Isaac, and Jacqueline Hanley

The Hospital for Sick Children, Anesthesia and Pain Medicine, Toronto, Ontario, Canada

The Sick Kids Transitional Pain Clinic was developed in 2011 and is the first of its kind in Canada. The goal of the clinic is to provide ongoing pain assessment/management to children and families after discharge from hospital to home or rehabilitation facility. The Transitional Pain Clinic bridges care following complex acute pain management or emergency services in hospital and provides early identification of those patients at risk for chronic pain.

Patients frequently have difficulty with pain management after discharge from hospital following surgery (Holland et al., 2011). The target population are children requiring high dose opioids, those reporting high pain scores, and those having difficulty returning to baseline function due to pain. Post-operative pain management can be a challenging task for families once home (Longard et al., 2016). The Transitional Pain Clinic addresses five main areas of need; weaning from opioids, titration of medication for complex pain; support of non-pharmacological pain management strategies; pain education needs at home and in the community; and early identification of those at risk for chronic pain. Referrals to the clinic continue to increase, as total visits (new referrals and follow-ups) are up 80% since the start of the clinic.

The clinic's structure and mandate, referral patterns, utilization statistics, a case study, and future directions including the use of web-based technology and research opportunities will be presented

**CONTACT** Lorraine Bird Lorraine.bird@sickkids.ca The Hospital for Sick Children, Anesthesia and Pain Medicine, Toronto, Ontario, Canada

© 2018 Lorraine Bird, Lisa Isaac and Jacqueline Hanley. Published with license by Taylor & Francis Group, LLC.
This is an Open Access article distributed under the terms of the Creative Commons Attribution License (http://creativecommons.org/licenses/by/4.0/), which permits unrestricted use, distribution, and reproduction in any medium, provided the original work is properly cited

## Engaging young adults in healthcare: the development of a young adult pain clinic

Sarah Sheffe[a] and Tania Di Renna[b]

[a]Toronto Academic Pain Medicine Institute, Women's College Hospital, Toronto, Canada; [b]Toronto Academic Pain Medicine Institute, Medical Director, Women's College Hospital Pain Clinic, Women's College Hospital, Toronto, Canada

Health care transition is the coordinated transfer of care from pediatric-oriented to adult-oriented health care for adolescents and young adults (AYA) with chronic medical conditions, leading to secure attachment to adult health services. [1] Transition programs are a key component of healthcare for this age group, and take into account the ongoing brain development and unique needs of AYA. [1] The Toronto Academic Pain Medicine Institute (TAPMI) Young Adult Clinic was developed to help fill this gap in pain care in Toronto, and to offer transition services for clients aged 17–25 with persistent pain to promote successful health care transition. The clinic was developed by TAPMI with consultation from a pediatric chronic pain clinic and pediatric transition program. Theory of Change was used as a framework to guide clinic development. The service evaluation strategy included clients, families, and clinicians, and an ongoing needs questionnaire is used to inform iterative development. Client outcomes measured include self-efficacy, pain acceptance, and goal attainment. A plan for clinic evaluation includes measurement of client visits and volumes, wait times, and secure attachment to adult services (i.e., attendance at initial appointment). This poster showcases best practice guidelines and strategies for program development and engagement of AYA clients through the example of the TAPMI Young Adult Clinic project development process.

**CONTACT** Sarah Sheffe sarah.sheffe@wchospital.ca

© 2018 Sarah Sheffe, and Tania Di Renna. Published with license by Taylor & Francis Group, LLC.
This is an Open Access article distributed under the terms of the Creative Commons Attribution License (http://creativecommons.org/licenses/by/4.0/), which permits unrestricted use, distribution, and reproduction in any medium, provided the original work is properly cited.

## Addressing beneath the surface of pain issues in rare genetic disorders – The Ehlers-Danlos Syndrome pain service- a novel initiative

Nimish Mittal, John Flannery, and Andrea Furlan

Department of Medicine – Division of Physical Medicine and Rehabilitation, University of Toronto and University Health Network, Toronto, Ontario, Canada

**Needs assessment**: Generalized musculoskeletal pain is the most common presentation in patients with Ehlers-Danlos Syndrome (EDS), a complex multisystem disorder caused by defects in collagen metabolis.[1] Due to limited knowledge of this disease, the pathway of care of EDS patients remains fragmented with poor access to appropriate services, yet, these patients have history of multiple visits to emergency departments and hospitalizations over the course of their lives. Many patients are prescribed extremely high dose of opioids, despite no evidence of opioid effectiveness in EDS.

**EDS pain services-UHN in Toronto**: The EDS pain service started in March 2017 at Toronto Rehabilitation Institute as a pilot project funded by a donation to the hospital's foundation. The clinic provides comprehensive interdisciplinary service involving three rehabilitation pain physicians, occupational therapist, physiotherapist, psychologist, pharmacist and social worker, and it has successfully provided pain rehabilitation consults to 50 patients since its inception. A chart review for descriptive analyses of the first 50 cases is currently underway. The clinic has successfully helped a number of patients on high opioid medication dose (between 200 and 1600 mg of morphine equivalent per day) to taper their opioid dose (median dose <50 mg per day) and manage pain with alternate strategies. Further, the clinic provides primary care providers with recommendations to help patients with EDS which include information on local self- management pain programs, and continuing medical education such as ECHO Ontario for chronic pain and opioid stewardship.

**CONTACT** Nimish Mittal nimish.mittal@uhn.ca Department of Medicine – Division of Physical Medicine and Rehabilitation, University of Toronto and University Health Network, Toronto, Ontario, Canada

© 2018 Nimish Mittal, John Flannery and Andrea Furlan. Published with license by Taylor & Francis Group, LLC.
This is an Open Access article distributed under the terms of the Creative Commons Attribution License (http://creativecommons.org/licenses/by/4.0/), which permits unrestricted use, distribution, and reproduction in any medium, provided the original work is properly cited.

## Development of an interprofessional clinical placement in a teaching-hospital pain clinic

Anne Marie Pinard[a], Isabelle Savard[b], Emmanuelle Careau[c], and Kadija Perreault[c]

[a]Faculty of Medicine, Anesthesiology and Intensive care Department, Centre Hospitalier Universitaire de Québec, Université Laval, Québec City, Québec, Canada; [b]Faculty of Medicine, Vice-Décanat à la pédagogie et au développement professionnel continu (VDPDPC), Laval University, Québec city, Québec, Canada; [c]Faculty of Medicine, Department of Rehabilitation, Centre for Interdisciplinary Research in Rehabilitation and Social Integration, Université Laval, Québec City, Québec, Canada

**Purpose**: Interprofessionnal care is at the heart of chronic pain management, but requires specific competencies. Preparing future healthcare providers to work collaboratively is essential. The aim of this project is to describe the development of an interprofessional clinical placement in a teaching-hospital pain clinic in Québec, Canada.

**Methods**: We used a pedagogical design approach to develop this interprofessional clinical placement project aiming to regroup students from physiotherapy, occupational therapy, nursing, psychology and pharmacy undergraduate programs, as well as chronic pain fellows (anaesthesia), from Université Laval. We undertook semi-structured interviews with the persons responsible for clinical placements in each program, analyzed program documents and professional competency profiles, and explored feasibility, barriers and facilitators, and the development of common interprofessional competency indicators through three focus groups with program representatives, the interprofessional team of pain clinic professionals and students. We used qualitative content analysis.

**Results**: Important deliverables include the construction and initial validation on an interprofessional competency evaluation scale for use in the pain clinic, and an online placement website for students and clinical supervisors. This work also highlights barriers and facilitators to the implementation of the placement, including difficulties in coordinating placements between programs and ensuring students have the necessary background. The expertise of professionals within the pain clinic was seen as a facilitator.

**Conclusions**: The actualization of interprofessional care requires appropriate training. Our work can inform academic and healthcare institutions wishing to develop Interprofessional clinical placements in contexts of chronic pain management.

**CONTACT** Anne Marie Pinard anne-marie.pinard@fmed.ulaval.ca

© 2018 Anne Marie Pinard, Isabelle Savard, Emmanuelle Careau and Kadija Perreault. Published with license by Taylor & Francis Group, LLC.
This is an Open Access article distributed under the terms of the Creative Commons Attribution License (http://creativecommons.org/licenses/by/4.0/), which permits unrestricted use, distribution, and reproduction in any medium, provided the original work is properly cited.

## Usability testing of a web-based resource to improve infant pain practices: the Implementation of Infant Pain Practice Change (ImPaC) resource

Mariana Bueno [a], Shirine Riahi[a], Navreet Gill[a], Shelly-Anne Li[b], Andy Willan[a], Anne Synnes[c], Carole Estabrooks[d], Christine Chambers [e], Denise Harrison [f], Janet Yamada[g], Jennifer Stinson[a], Jim Slotta[h], Marsha Campbell-Yeo[i], Melanie Barwick [j], Melanie Noel[k], Sharyn Gibbins[l], Sylvie LeMay[m], Wanrudee Isaranuwatchai[n], and Bonnie Stevens[o,p]

[a]Child Health Evaluative Sciences, The Hospital for Sick Children, Toronto, Ontario, Canada; [b]Nursing, University of Toronto, Toronto, Ontario, Canada; [c]Pediatrics, University of British Columbia, Vancouver, British Columbia, Canada; [d]Nursing, University of Alberta, Edmonton, Alberta, Canada; [e]Pediatrics, Dalhousie University, Halifax, Nova Scotia, Canada; [f]University of Ottawa, Health Sciences, and Children's Hospital of Eastern Ontario, Ottawa, Ontario, Canada; [g]Nursing, Ryerson University, Toronto, Ontario, Canada; [h]Curriculum, Teaching and Learning, University of Toronto, Toronto, Ontario, Canada; [i]School of Nursing, Dalhousie University, Halifax, Nova Scotia, Canada; [j]Psychiatry, The Hospital for Sick Children, Toronto, Ontario, Canada; [k]Psychology, University of Calgary, Calgary, Alberta, Canada; [l]Professional Practice, Trillium Health Partners, Mississauga, Ontario, Canada; [m]Nursing, Université de Montréal, Montreal, Quebec, Canada; [n]Centre for Excellence in Economic Analysis Research, St. Michael's Hospital, Toronto, Ontario, Canada; [o]Lawrence S Bloomberg Faculty of Nursing, University of Toronto, Toronto, Ontario, Canada; [p]Senior Scientist Research Institute, The Hospital for Sick Children, Toronto, Ontario, Canada

**Introduction/Aim**: the Implementation of Infant Pain Practice Change (ImPaC) Resource is a web-based tool for guiding health care professionals (HCPs) through a practice change process to improve infant pain assessment and treatment. Our aim was to test the usability of the ImPaC Resource prototype with end-users.

**Methods**: HCPs from two units at one pediatric hospital participated in two waves (W1 & W2) of individual usability test sessions with a trained facilitator. Participants navigated through the Resource using a prescribed scenario while "thinking aloud". Sessions were audio and screen-recorded. A survey and semi-structured interview were administered; qualitative and quantitative analyses were performed.

**Results**: 10 HCPs completed W1 (n = 5) and W2 (n = 5) of usability testing. In both W1 and W2, participants were satisfied or very satisfied (40% and 60%) with the Resource, and considered it easy or very easy to understand (60% and 20%). Median System Usability Scale (SUS) scores were 70 (65–87.5) in W1 and 77.5 (52.5–92.5) in W2. After W1, suggested changes provided by ≥2 users were analyzed and incorporated into the prototype. Minor technical errors were corrected. No further changes were suggested following W2. Users in both waves considered the Resource as intuitive and easy to navigate (60%), and visually appealing (80%) with trustworthy content (80%). Most users agreed the Resource would be useful to guide pain practice changes in their units (80%).

**Conclusions**: the Resource was considered satisfying and easy to understand. Good usability scores (≥70) were achieved. The ImPaC Resource will next be tested on a clinical unit.

**CONTACT** Mariana Bueno mariana.bueno@sickkids.ca

© 2018 Mariana Bueno, Shirine Riahi, Navreet Gill, Shelly-Anne Li, Andy Willan, Anne Synnes, Carole Estabrooks, Christine Chambers, Denise Harrison, Janet Yamada, Jennifer Stinson, Jim Slotta, Marsha Campbell-Yeo, Melanie Barwick, Melanie Noel, Sharyn Gibbins, Sylvie LeMay, Wanrudee Isaranuwatchai and Bonnie Stevens. Published with license by Taylor & Francis Group, LLC.
This is an Open Access article distributed under the terms of the Creative Commons Attribution License (http://creativecommons.org/licenses/by/4.0/), which permits unrestricted use, distribution, and reproduction in any medium, provided the original work is properly cited.

## Using technology to help clinicians and patients manage pain

Salima S. J. Ladak[a], Abid Azam[b], Tahir Janmohamed[c], Karim Ladha[d], Diana Tamir[d], Aliza Weinrib[d], Rita Katznelson[d], Joel Katz[e], and Hance Clarke[f]

[a]Toronto General Hospital, Anesthesia and Pain Management, Toronto, ONT, Canada; [b]York University, Department of Psychology, Toronto, ONT, Canada; [c]Founder of ManagingLife, Toronto, ONT, Canada; [d]Toronto General Hospital, Anesthesia and Pain Management, Toronto, ONT, Canada; [e]Department of Psychology, York University, Toronto, ONT, Canada; [f]Department Anesthesia and Pain Management, University Health Network, Toronto, ONT, Canada

Continuity of pain management care following hospital discharge from painful interventions continues to be problematic. From the time of hospital discharge to the next outpatient appointment in a pain clinic, patients often cannot recall pain qualities or patterns required for accurate assessment and optimal treatment. To address this gap, an innovative mobile and web-based application (app) has been developed that enables patients to document their daily activities and pain experiences in less than 60 seconds, and allows them to monitor their symptom patterns and treatment progress over time. This app serves as a clinical and educational tool between and prior to pain treatment appointments, enabling clinicians of our Transitional Pain Service to review pain and associated physical and psychological functional patterns. An intended educational benefit for patients is to use the app's features including visuals such as graphs and charts, to better understand the nature, pattern, and triggers of their pain on a day-to-day basis. Since its pilot deployment at the Toronto General Hospital, 34 patients have used the app for self-management. Over 90% of patient-users in a clinical trial reported that the app would make it easier for them to communicate with their clinician. The app provides summary reports of key pain patterns (e.g. intensity, duration, time of pain event), associated levels of physical function as well as mood, all of which are used during follow up transitional pain clinic appointments. This poster illustrates clinical and educational features of the *Manage My Pain* app.

**CONTACT** Salima S. J. Ladak Salima.Ladak@uhn.ca

© 2018 Salima S. J. Ladak, Abid Azam, Tahir Janmohamed, Karim Ladha, Diana Tamir, Aliza Weinrib, Rita Katznelson, Joel Katz and Hance Clarke. Published with license by Taylor & Francis Group, LLC.
This is an Open Access article distributed under the terms of the Creative Commons Attribution License (http://creativecommons.org/licenses/by/4.0/), which permits unrestricted use, distribution, and reproduction in any medium, provided the original work is properly cited.

## Implementation of a Humanoid Robot to Reduce Procedural Pain and Distress in a Pediatric Blood Collection Lab: Facilitators and Barriers

LeAnne Revell[a], Christine T. Chambers[b], Jennifer Parker[c], Leota Dickey[d], Patricia Collins[d], Chantal LeBlanc[e], Katelynn Boerner[f], Larson Alfhild[g], and Heather McNeil[h]

[a]Centre for Pediatric Pain Research and Medicine, IWK Health Centre and Dalhousie UniversityHalifax, Nova Scotia, Canada; [b]Centre for Pediatric Pain Research and Dalhousie Departments of Psychology and Pediatrics, IWK Health Centre and Dalhousie University, Halifax, Nova Scotia, Canada; [c]Centre for Pediatric Pain Research and Dalhousie Department of Pediatrics, IWK Health Centre and Dalhousie Univeristy, Halifax, Nova Scotia, Canada; [d]Pathology and Laboratory Medicine, IWK Health Centre, Halifax, Nova Scotia, Canada; [e]Child Life Services, IWK Health Centre, Halifax, Nova Scotia, Canada; [f]Clinical Psychology, Dalhousie University, Halifax, Nova Scotia, Canada; [g]Department of General Pediatrics, IWK Health Centre, Nova Scotia, Canada; [h]Pathology of Laboratory Medicine, IWK Health Centre, Nova Scotia, Canada

**Introduction:** Venipuncture is an important procedure in children's healthcare, but unfortunately it can cause substantial pain and distress for children and their families. Controlled Studies have demonstrated that the use of a humanoid robot, MEDi®, can reduce pain and distress experienced by children and families during medical procedures. The aim of this study was to identify parent, child, and hospital staff perspectives on potential facilitators and barriers of implementing a humanoid robot into the blood collection lab in a pediatric hospital.

**Methods:** Eight semi-structured focus groups/interviews were held with a total of 30 participants. Two were with hospital staff (e.g. child life specialists, phlebotomists), one with undergraduate volunteers and five with children and their parents. Sessions included discussion and interaction with the robot, which was video recorded. Deductive thematic analysis was completed with NVivo® qualitative data software and guided by the Consolidated Framework for Implementation Research.

**Results:** Facilitators identified were positive receptivity, the need for intervention, and the novelty of MEDi® compared to other distraction techniques. Barriers identified were volunteer training and infrastructure limitations.

**Conclusions:** These findings will be used to inform the introduction of the robot into a clinical setting in order to optimize implementation effectiveness.

**CONTACT** LeAnne Revell leanne.revell@dal.ca

© 2018 LeAnne Revell, Christine T. Chambers, Jennifer Parker, Leota Dickey, Patricia Collins, Chantal LeBlanc, Katelynn Boerner, Larson Alfhild and Heather McNeil. Published with license by Taylor & Francis Group, LLC.
This is an Open Access article distributed under the terms of the Creative Commons Attribution License (http://creativecommons.org/licenses/by/4.0/), which permits unrestricted use, distribution, and reproduction in any medium, provided the original work is properly cited.

### A survey of healthcare practitioners' knowledge of myofascial pain syndrome criteria

Sara Ahmed[a], Yasmin Nasirzadeh[b], Sasha Monteiro[c], John Srbley[c], and Dinesh Kumbhare[d]

[a]Department of Psychology, McMaster University, Hamilton, Ontario, Canada; [b]Medicine, University of Toronto, Toronto, Ontario, Canada; [c]University of Guelph, Guelph, Ontario, Canada; [d]Medicine, Toronto Rehabilitation Institute, Toronto, Ontario, Canada

**Introduction**: The goal of this study was to assess agreement on the signs and symptoms of myofascial pain for chiropractors, physicians, and registered massage therapists (RMT).

**Methods**: 337 healthcare practitioners participated in the survey. The questionnaire probed clinician agreement with a set of signs and symptoms using a seven-point agreement scale (1-absolutely agree, 7-absolutely disagree). Agreement was assessed using intraclass correlation analyses within and between all study groups. Descriptive statistics, including mean response values, were used to assess which signs or symptoms were most often associated with myofascial pain.

**Results**: There was poor agreement among chiropractors, physicians, and RMTs on the signs and symptoms that represent myofascial pain syndrome (ICC = 0.59). Physicians and RMTs were in agreement on four statements: point tenderness on a taut band of muscle would be present, a local twitch response occurring after palpation of painful areas of muscle, the presence of referred pain after palpating painful areas, and the reproduction of usual pain after repeated palpation of the painful area. Chiropractors were in agreement on a different set of signs and symptoms relative to physicians and RMTs, and expressed neutrality on most statements. RMTs had the most within-group agreement (ICC = 0.80) relative to chiropractors (ICC = 0.59) and physicians (ICC = 0.51).

**Discussion**: Our results suggest that there is a lack of agreement within and between healthcare practitioner groups on the signs and symptoms that define myofascial pain syndrome. We suggest the demonstrated variability in diagnostic knowledge be remedied through the establishment and use of official validated criteria.

**Conclusion**: The quantitative results of this study suggest that patients overall experience more subjective pain relief with local analgesic injections. Further research is required to determine sources of heterogeneity contributing to the differences found between studies assessing the effectiveness of local analgesic and BTX-A injections.

**CONTACT** Sara Ahmed saraahmed1510@gmail.com

© 2018 Yasmin Nasirzadeh, Sara Ahmed, Sasha Monteiro, John Srbley and Dinesh Kumbhare. Published with license by Taylor & Francis Group, LLC.
This is an Open Access article distributed under the terms of the Creative Commons Attribution License (http://creativecommons.org/licenses/by/4.0/), which permits unrestricted use, distribution, and reproduction in any medium, provided the original work is properly cited.

## Piloting a transitional pain program in kingston for postsurgical patients: implementation of RNAO best practice guidelines for pain

Samantha Kim[a], Mona Sawhney[b], Scott Duggan[c], John Murdoch[d], and Sue Vasily[e]

[a]Kingston General Hospital, Acute Pain Management Services, Kingston, Ontario, Canada; [b]Nursing, Kingston, Queen's University, Kingston, Ontario, Canada; [c]Chronic Pain Clinic, Hotel Dieu Hospital, Kingston, Ontario, Canada; [d]Acute Pain Management Services, Kingston General Hospital, Kingston, Ontario, Canada; [e]Acute Pain Management Services, Kingston General Hospital, Kingston, Ontario, Canada

Moderate-to-severe chronic post-surgical pain (CPSP) occurs in approximately 10% of patients after major surgery and has an annual cost of $41,000 per patient in the US. The global annual cost of CPSP is in the hundreds of billions of dollars.[1] Kingston, Ontario currently lacks services specifically aimed at minimizing a post-surgical patient's development of CPSP and limited community resources are available to treat existing CPSP. The Registered Nurses Association of Ontario's Advanced Clinical Practice Fellowship Program provided an avenue to address this gap through implementation of Best Practice Guidelines.

A modified version of the established Transitional Pain Service (TPS) program at Toronto General Hospital (TGH) was piloted within the Kingston Health Sciences Centre (KHSC) between October 2017 and February 2018. Similar to TGH's TPS clinic, KHSC's utilized a multidisciplinary team approach. Potential TPS candidates were identified and recruited. These patients received pain education prior to discharge and were followed at the TPS clinic as outpatients for 8 weeks. TPS staff worked closely with patients' primary care providers (PCP); a patient's pain management was transferred back to their PCPs when their pain level, functioning and pain medication doses were back to baseline pre-operatively. Evaluation of the program was completed through patient surveys. In the future, the goal includes expanding upon the clinic by recruiting patients in the pre-admission phase and focusing on a specific patient population who will benefit from this service. This presentation will discuss the key factors in creating this program and patient outcomes.

**CONTACT** Samantha Kim Samantha.Kim@kingstonhsc.ca

© 2018 Samantha Kima, Mona Sawhneyb, Scott Dugganc, John Murdochd and Sue Vasilye. Published with license by Taylor & Francis Group, LLC.
This is an Open Access article distributed under the terms of the Creative Commons Attribution License (http://creativecommons.org/licenses/by/4.0/), which permits unrestricted use, distribution, and reproduction in any medium, provided the original work is properly cited.

## Rapid access to interprofessional pain management using a stepped care model

Patricia A. Poulin[a,b], Cristin Kargus[c], Renée Gauthier[c], Anne-Rose Robbins[c], and Susan Ward[c]

[a]The Ottawa Hospital Research Institute, The Ottawa Hospital Pain Clinic, Ottawa, ON, Canada; [b]School of Psychology, Faculty of Social Sciences and Department of Anesthesiology, Faculty of Medicine, University of Ottawa, Ottawa, ON, Canada; [c]The Ottawa Hospital Pain Clinic, Ottawa, ON, Canada

Wait-time for interdisciplinary pain programs across Canada varies between 6 months and 5 years. It is well-established that people living with chronic pain who are waiting for more than 6 months for tertiary pain care experience significant deterioration in function, mood, and quality of life. In addition, many patients can never access care due to geographical, physical or cultural barriers or because they do not meet the programs' entry criteria. Innovative approaches to increase accessibility and reduce wait-time are needed. Stepped care models have been applied to the field of mental health and may provide an interesting avenue to explore. The Ottawa Hospital Pain Clinic Interprofessional Team (social work, occupational therapy, physiotherapy, and psychology) was established in September 2016 and quickly encountered growing wait-times. Using a participatory action research framework, the team developed a stepped care approach providing rapid (<2 weeks) access to educational pain-self management strategies delivered in small group settings and a personalized treatment plan within 1 month from referral. Before program implementation, wait-times to access therapists or interdisciplinary care was greater than 6 months. After implementing the program, more than 90% of all consultation requests to the team were responded to within a 2 week period. The flexibility of the care model also allows for cross coverage ensuring that all patients are seen for assessment and personalized treatment leveraging all resources available. A stepped care approach to chronic pain management is a viable and cost-effective approach for pain centers facing long wait-times for inter-disciplinary care.

**CONTACT** Patricia A. Poulin ppoulin@toh.ca The Ottawa Hospital, Psychology, Ottawa, ON, Canada

© 2018 Patricia A. Poulin, Cristin Kargus, Renée Gauthier, Rose Robbins and Susan Ward. Published with license by Taylor & Francis Group, LLC.
This is an Open Access article distributed under the terms of the Creative Commons Attribution License (http://creativecommons.org/licenses/by/4.0/), which permits unrestricted use, distribution, and reproduction in any medium, provided the original work is properly cited.

### Low Dose Naltrexone (LDN) in the treatment of neuropathic pain secondary to chronic lyme disease

Kevin Kordbacheh[a], Stefan Marković[b], Karina Liubchenko[c], Tanja Visnjevac[d], and Ognjen Visnjevac[e]

[a]Human Biology, University of Toronto, Toronto, Ontario, Canada; [b]St. George's University School of Medicine, Bay Shore, NY, USA; [c]Physiology and Human Biology, University of Toronto, Toronto, Ontario, Canada; [d]Toronto, Ontario, Canada; [e]Faculty of Health Sciences, McMaster University Department of Anesthesiology, Ontario, Canada

Although naltrexone has classically been used as a deterrent for opioid and alcohol abuse during rehabilitation, in recent years it has had an increasing number of applications in chronic pain management at low doses (LDN). At 1-5mg per day, it has been reported to treat pain secondary to fibromyalgia, Crohn's disease, complex regional pain syndrome, cancer pain, and diabetic neuropathy. We report the first case of naltrexone use for the treatment of neuropathic pain secondary to chronic Lyme disease, with improvement in hypoesthesia and sensory deficits, along with a 6$^{th}$ cranial nerve palsy, for a 3 month period since initiation of therapy (and ongoing). Due to improvements in 6$^{th}$ cranial nerve palsy, the patient reported improvements in double vision, balance, and gait, correlating with objective observations of right eye abduction normalization, which was also corroborated by observations of family members.

Although chronic Lyme disease is normally treated with long-term antibiotics such as doxycycline or azithromycin, this is not always successful. In this case report, we describe a previously unpublished treatment modality, LDN, which highlights an opportunity to study this medication as an adjunct to antibiotics, as well as an alternative to chronic antibiotic therapy. Further study is, of course, needed to assess outcomes in a more robust sample before any generalizable conclusions can be drawn.

**CONTACT** Kevin Kordbacheh kevin.kordbacheh@mail.utoronto.ca

© 2018 Kevin Kordbacheh, Stefan Marković, Karina Liubchenko, Tanja Visnjevac and Ognjen Visnjevac. Published with license by Taylor & Francis Group, LLC.
This is an Open Access article distributed under the terms of the Creative Commons Attribution License (http://creativecommons.org/licenses/by/4.0/), which permits unrestricted use, distribution, and reproduction in any medium, provided the original work is properly cited.

## Implementing a 12-session interdisciplinary chronic pain self-management program at Kingston Health Sciences Centre-Hotel Dieu Hospital site in Kingston, Ontario

Kyle Vader[a], Elizabeth Brown[a], Tom Doulas[a], Mary Anne Good[a], Rebecca McDermott[a], Kerry Oulton[a], Christine Varey[a], Scott Duggan[a], and Catherine Donnelly[b]

[a]Kingston Health Sciences Centre-Hotel Dieu Hospital site, Chronic Pain Clinic, Kingston, Ontario, Canada; [b]School of Rehabilitation Therapy, Queen's University, Kingston, Ontario, Canada

**Background**: In consultation with the School of Rehabilitation Therapy at Queen's University, the Chronic Pain Clinic at Kingston Health Sciences Centre-Hotel Dieu Hospital site has developed and is in the process of implementing a 12-session interdisciplinary chronic pain self-management program.

**Implementation**: Since September 2016, five cohorts of patients have participated in the program. Adherence and attendance has proven to be a challenge for some patients. As a result, clearly defined inclusion and exclusion criteria are being developed.

**Structure**: Before entering the program, potential participants engage in a novel 'pre-group assessment' with a physiotherapist and occupational therapist to determine their readiness to engage. The first 10 sessions of the program are delivered in a hospital conference room before transitioning to a community-based facility for the final two sessions. After the program, participants complete a 'post-group evaluation' with a healthcare provider to reflect on their experiences and plan for the future. The program emphasizes non-pharmacological pain management strategies and is delivered collaboratively by occupational therapy, physiotherapy, and psychology. Specific teaching is also provided by nursing and medicine.

**Measurement**: Validated outcome measures are implemented pre-, post-, as well three months following completion of the program. Outcome measures include the Tampa Scale for Kinesiophobia, Brief Pain Inventory-Short Form, Pain Stages of Change Questionnaire, Canadian Occupational Performance Measure, Pain Catastrophizing Scale, Short Musculoskeletal Functional Assessment, Pain Self-Efficacy Questionnaire, and a satisfaction questionnaire.

**Next Steps**: Next steps include a formalized program evaluation including outcomes and perspectives from patients, healthcare providers, hospital administrators, and community agencies.

**CONTACT** kyle.vader kyle.vader@KingstonHSC.ca Kingston Health Sciences Centre-Hotel Dieu Hospital site, Chronic Pain Clinic, Kingston, Ontario, Canada

© 2018 Kyle Vader, Elizabeth Brown, Tom Doulas, Mary Anne Good, Rebecca McDermott, Kerry Oulton, Christine Varey, Scott Duggan and Catherine Donnelly. Published with license by Taylor & Francis Group, LLC.
This is an Open Access article distributed under the terms of the Creative Commons Attribution License (http://creativecommons.org/licenses/by/4.0/), which permits unrestricted use, distribution, and reproduction in any medium, provided the original work is properly cited.

## A smartphone-based mindfulness intervention for chronic pain

Vered Valeria Latman[a], Muhammad Abid Azam[a], Arielle Sutton[b], Amir Zarie[a], Helia Ghazinejad[a], and Joel Katz[a]

[a]Psychology, York University, Toronto, Ontario, Canada; [b]Psychology, McGill University, Toronto, Ontario, Canada

**Introduction**: We developed a novel 12-minute smartphone-based mindfulness application for symptoms of anxiety and tested it in four groups of university students: n = 42 with chronic pain (CP), n = 39 with symptoms of depression/anxiety (DA), and condition-free controls with (CF+; n = 54) and without (CF-; n = 26) a smartphone application.

**Methods**: Participants completed the Tension-Anxiety subscale of the Profile of Mood States at baseline (pre) and post-intervention. Participants were instructed on the smartphone-based Breath Awareness Task, which involves paying attention to the flow of breath and pressing "breath" or "other" buttons on a smartphone at the sound of a tone (presented randomly 24 times over 12 minutes). The CF- group attended to their breath without use of the smartphone app.

**Results**: We used a 2-way mixed factor ANOVA with Time (baseline, post intervention) and Group (CP, DA, CP+, CP-) as factors to evaluate changes in Tension-Anxiety scores. The simple main effect of Group was significant at baseline ($p < .001$, $\eta p2 = .27$) and post-intervention ($p < .001$, $\eta p2 = .28$). At baseline, DA and CP groups had similarly high levels of Tension-Anxiety, which were significantly higher than both CP+ and CP- ($p < .001$). Post-intervention, Tension-Anxiety scores in the CP group dropped to a level similar to that of the condition-free groups, while the DA group remained significantly higher than the 3 other groups ($p < .001$).

**Discussion**: The 12-minute mindful breathing app reduced Tension-Anxiety scores in participants with chronic pain. These findings are particularly meaningful given the high prevalence of anxiety symptoms in individuals with chronic pain and the role of anxiety in pain perception and coping.

**CONTACT** Vered Latman vvlatman@yorku.ca

© 2018 Vered Valeria Latman, Muhammad Abid Azam, Arielle Sutton, Amir Zarie, Helia Ghazinejad and Joel Katz. Published with license by Taylor & Francis Group, LLC.
This is an Open Access article distributed under the terms of the Creative Commons Attribution License (http://creativecommons.org/licenses/by/4.0/), which permits unrestricted use, distribution, and reproduction in any medium, provided the original work is properly cited.

### Science of pain: an innovative model for pain neuroscience education in pediatrics

Katelyn Wilkinson and Elana Jackson

Pediatric Chronic Pain Program, McMaster Children's Hospital, Hamilton, Ontario, Canada

**Background**: Pain Neuroscience Education (PNE) is an educational model designed to help patients reconceptualise pain and its function. In adult chronic pain patients, varied forms of this intervention have been associated with reductions in pain ratings, disability, pain catastrophizing, and fear-avoidanc.[1] Several delivery models for PNE have been described and evaluated with adult chronic pain populations. Limited research has been done with adolescent populations and to our knowledge, no standardized, empirically evaluated patient curriculum for pediatric PNE currently exists.

**Innovation**: We developed and implemented a standardized pediatric PNE session using a continuous quality improvement process. Currently, we offer a one hour "Science of Pain" session facilitated by a Nurse Practitioner and Child Life Specialist to new patients and their families. This stand-alone session is child-focused, interactive, family-centred, and individualized to each patient's developmental needs and pain story.

**Impact**: Since March 2017, 92 patients have received the session (82% of all new intakes). Initial feedback from staff, patients, and families has been overwhelmingly positive. There is significant interest from the pediatric pain community and health care providers working in other clinical areas. Future directions include manualizing the session protocol for dissemination, conducting feasibility testing, and gathering formal feedback regarding patient satisfaction and knowledge retention.

**CONTACT** Katelyn Wilkinson wilkinkate@hhsc.ca

© 2018 Katelyn Wilkinson and Elana Jackson. Published with license by Taylor & Francis Group, LLC.
This is an Open Access article distributed under the terms of the Creative Commons Attribution License (http://creativecommons.org/licenses/by/4.0/), which permits unrestricted use, distribution, and reproduction in any medium, provided the original work is properly cited.

## Effectiveness of Local Anesthetic versus Botulinum Toxin Injections for Myofascial Pain: A Systematic Review and Meta-analysis

Sara Ahmed[a], Shoba Subramaniam[b], Shereen Khattab[c], Kamaldeep Sidhu[d], Dhanveer Singh[d], and Dinesh Kumbhare[b]

[a]Department of Psychology, McMaster University, Hamilton, Canada; [b]Medicine, Toronto Rehabilitation Institute, Toronto, Canada; [c]Department of Kinesiology, McMaster University, Hamilton, Canada; [d]Science, University of Western Ontario, London, Canada

**Introduction**: Myofascial pain is a chronic pain disorder characterized by the presence of painful localized regions of muscle and myofascial trigger points (MTrP). MTrPs are bands of skeletal muscle that are stiff and hypersensitive on palpation. Intramuscular MTrP injections are considered first-line treatments for myofascial pain. Injectates can include local anesthetic and Botulinum toxin-A (BTX-A). The objective of this systematic review was to compare the effectiveness of local analgesics and BTX-A on reported pain in patients with myofascial pain.

**Methods**: A comprehensive systematic search of three databases, EMBASE, Cochrane CENTRAL, and Medline. The search was comprised of words to describe the following key terms: “myofascial pain”, “injections”. We performed meta-analyses comparing local anesthetic and BTX-A injections across these follow-up week periods: 0, 1–2, 3–4, 8–9, 11–12, 16, 24. The extracted outcome measure was subjective pain intensity.

**Results**: Thirty-four studies were included. Qualitative analysis suggests that local anesthetic and BTX-A are not consistently effective at mitigating pain across all follow-up periods. The meta-analyses revealed local anesthetic injections are more effective (effect size favored the treatment) over all follow-up periods, except 0 and 11–12 weeks, for mitigating pain intensity compared to BTX-A injections, which were not effective at reducing pain intensity except at 24 weeks.

**Conclusions**: Additional studies are needed to determine sources of heterogeneity resulting in the differences found between studies on the effectiveness of local anesthetic and BTX-A injections. The quantitative results of this study suggest that patients overall experience more subjective pain relief with local anesthetic injections.

**CONTACT** Sara Ahmed saraahmed1510@gmail.com

© 2018 Sara Ahmed, Shoba Subramaniam, Shereen Khattab, Kamaldeep Sidhu, Dhanveer Singh and Dinesh Kumbhare. Published with license by Taylor & Francis Group, LLC.
This is an Open Access article distributed under the terms of the Creative Commons Attribution License (http://creativecommons.org/licenses/by/4.0/), which permits unrestricted use, distribution, and reproduction in any medium, provided the original work is properly cited.

## Demographics and outcomes of chronic pain patients treated by a publically funded interdisciplinary community pain program in Ontario

Angela Mailis[a,b,c], Karen Spivak[d], and Shah Fatima Lakha[e]

[a]Pain and Wellness Centre, Vaughan, Ontario, Canada; [b]Dept. of Medicine, Division of Physical Medicine, UofToronto, Canada; [c]Toronto Rehab Institute/University Health Network, Toronto, Ontario, Canada; [d]Pain and Wellness Centre, Psychology, Vaughan, Ontario, Canada; [e]Pain and Wellness Centre, Vaughan, Ontario, Canada

**Aim:** To present demographic and outcome data of selected patients treated by a community-based interdisciplinary chronic pain team.

**Methods:** Demographics and validated questionnaire responses were collected from participants in a customized chronic pain treatment program linked to the Toronto Academic Pain Medicine Institute (TAPMI)-related hospitals and funded by the Ontario Ministry of Health and Long Term Care.

**Results:** During an 18-month period, we offered 565 new patient consultations and 1,141 follow up visits. Eligible patients within a 50km radius (N = 111) were admitted to our TAPMI program: 66 females, 45 males; mean age 45.1; 17% were >65;

41.4% at work; 34.2% had received injections in the past; 35% were current users of cannabis; 28.8% were involved in car accidents; 39.7% had multisite or widespread pain; with mean pain NRS 6.9 ± 1.47. Despite customized and charge-free interdisciplinary care, 19 participants (17%) dropped out or dismissed due to poor attendance/performance (11% of males and 21% of females, $p < 0.05$). Currently, 45 patients are still in treatment and 47 completed the program. Outcome data to date indicate substantial improvement in several scales (60.2% in self-efficacy, 48.8% in pain catastrophizing, 49.8% in GAD, 41.5% in BPI, 31.2% in CESD), with 83% of the participants much improved in Global Impression of Change. Detailed analysis and additional data will be presented.

**Conclusion:** Interdisciplinary pain management is not for everyone. Responsible utilization of taxpayer's money requires proper selection of participants as this seems to be a crucial factor in the success of such programs.

**CONTACT** Angela Mailis angela.mailis@uhn.ca Pain and Wellness Centre, Vaughan Ontario, Canada.

© 2018 Angela Mailis, Karen Spivak and Shah Fatima Lakha. Published with license by Taylor & Francis Group, LLC.
This is an Open Access article distributed under the terms of the Creative Commons Attribution License (http://creativecommons.org/licenses/by/4.0/), which permits unrestricted use, distribution, and reproduction in any medium, provided the original work is properly cited.

### Building sustainable partnerships with patients and families to guide pediatric chronic pain research and practice in Canada

Kathryn A. Birnie[a], Jennifer Stinson[a], Melanie Noel[b], Jill Chorney[c], Krista Baerg[d], Paula Forgeron[e], Christine Lamontagne[f], Patricia Poulin[g], Katherine Dib[h], Mary-Anne Dib[i], Carley Ouellette[j], Kimberly Nelson[k], Matthew Dawson[l], Bill Dawson[m], and Nikki Cooke[n]

[a]Lawrence S. Bloomberg Faculty of Nursing, University of Toronto & Child Health Evaluative Sciences, The Hospital for Sick Children, Toronto, Ontario, Canada; [b]Department of Psychology, University of Calgary, Calgary, Alberta, Canada; [c]Department of Anesthesia, Pain Management & Perioperative Medicine, Dalhousie University & IWK Health Centre, Halifax, Nova Scotia, Canada; [d]Department of Pediatrics, University of Saskatchewan & Royal University Hospital, Saskatoon, Saskatchewan, Canada; [e]School of Nursing, University of Ottawa, Ottawa, Ontario, Canada; [f]Department of Anesthesiology and Pain Medicine, University of Ottawa and Children's Hospital of Eastern Ontario, Ottawa, Ontario, Canada; [g]Department of Anesthesiology and Pain Medicine, University of Ottawa & The Ottawa Hospital, Ottawa, Ontario, Canada; [h]Patient Partner, Halifax, Nova Scotia, Canada; [i]Parent Partner, Halifax, Nova Scotia, Canada; [j]Patient Partner, Nursing, McMaster University, Hamilton, Ontario, Canada; [k]Parent Partner, Windsor, Ontario, Canada; [l]Patient Partner, Calgary, Alberta, Canada; [m]Parent Partner, Calgary, Alberta, Canada; [n]Patient Partner, Regina, Saskatchewan, Canada

**Objective**: The primary aim of this project is to co-build a sustainable patient engagement strategy to guide pediatric chronic pain research and practice in Canada. "*Patient engagement occurs when patients meaningfully and actively collaborate in the governance, priority setting, and conduct of research, as well as in summarizing, distributing, sharing, and applying its resulting knowledge*" (CIHR, 2014).

**Design**: This poster outlines ongoing activities of this patient engagement project, including plans to: 1) create a Canadian patient engagement registry of pediatric patients and families who are interested and able to act as partners and collaborators in research and clinical practice in pediatric chronic pain; 2) facilitate capacity building for pediatric patient engagement by implementing pediatric specific training for patients, families, clinicians, and researchers; and 3) co-build a national research agenda for pediatric chronic pain with patients, families, and clinicians from across Canada. Evaluation and knowledge dissemination is embedded throughout. Our national team is comprised of former pediatric chronic pain patients, parents, clinicians, researchers, policy-makers and advocacy groups that form a partnership of relevant engaged stakeholders.

**Conclusions**: Patient engagement involving children and families enhances the quality, appropriateness, and relevance across all stages of scientific knowledge. By enhancing pediatric patient and family engagement, we believe that research will be more effective, efficient, and clinically meaningful to pediatric patients and their parents. This will increase the likelihood that research will be taken up in clinical care, lead to better patient outcomes, and avoid wasting effort in areas that are not of importance to patients.

**CONTACT** Kathryn A. Birnie kathryn.birnie@sickkids.ca

© 2018 Kathryn A. Birnie, Jennifer Stinson, Melanie Noel, Jill Chorney, Krista Baerg, Paula Forgeron, Christine Lamontagne, Patricia Poulin, Katherine Dib, Mary-Anne Dib, Carley Ouellette, Kimberly Nelson, Matthew Dawson, Bill Dawson and Nikki Cooke. Published with license by Taylor & Francis Group, LLC.
This is an Open Access article distributed under the terms of the Creative Commons Attribution License (http://creativecommons.org/licenses/by/4.0/), which permits unrestricted use, distribution, and reproduction in any medium, provided the original work is properly cited.

### A community of practice in action: development of E-toolkits for minimizing pain for all children

Samina Ali[a], Ashleigh Townley[b], Kathy Reid[c], Sandy Smeenk[d], and Lisa Stromquist[e], and CAPHC Pain Community of Practice[,e]

[a]Pediatrics, University of Alberta, Edmonton, Canada; [b]Evidence to Care, Holland Bloorview Kids Rehabilitation Hospital, Toronto, Canada; [c]Pediatric Chronic Pain Service, Stollery Children's Hospital, Edmonton, Canada; [d]ILC Foundation, Toronto, Canada; [e]Canadian Association of Paediatric Health Centres, Ottawa, Canada

In 2012, the Canadian Association of Paediatric Health Centres (CAPHC) identified paediatric pain as a priority for a Community of Practice (CoP). Its goal was: to improve healthcare pain practices, quality and safety, building national collaborations between paediatric institutions, sharing experiences regarding the development, implementation and evaluation of paediatric pain practice guidelines. The target audience was CAPHC member institutions, reaching non-paediatric-focused centres.

CAPHC invited healthcare professionals interested in paediatric pain to join this CoP and identified three key areas of focus: procedural, acute, and chronic pain. Two surveys were conducted to determine current practice, knowledge gaps and barriers to practice change. To address the evidence to practice gap CoP members agreed to a compendium of best practice resources.

Relevant systematic reviews were appraised; it was determined that electronic toolkits (e-toolkits) would be developed. CoP members co-created content for the e-toolkits; collecting policy examples, educational presentations, family resources and videos, background articles, and other practical implementation tools. Fourteen CoP clinical experts assessed relevance and quality of content.

Three evidence-based open-access e-toolkits focused on procedural, acute, and chronic pain launched to the CAPHC community between June 2016 and December 2017. They are accessible through the CAPHC Knowledge Exchange Network and include: pain assessment, seven specific procedural pain interventions, three distinct areas of acute pain, general and specific chronic pain resources.

Future work will focus on toolkit dissemination, supporting CAPHC membership to use the toolkits, expanding the reach of these efforts to non-CAPHC members by partnering with other national knowledge mobilization efforts (TREKK.ca).

**CONTACT** Samina Ali sali@ualberta.ca

© 2018 Samina Ali, Ashleigh Townley, Kathy Reid and Sandy Smeenk, and Lisa Stromquist. Published with license by Taylor & Francis Group, LLC.
This is an Open Access article distributed under the terms of the Creative Commons Attribution License (http://creativecommons.org/licenses/by/4.0/), which permits unrestricted use, distribution, and reproduction in any medium, provided the original work is properly cited.

### Assessing usage, satisfaction, and effectiveness of a peer group for youth with chronic pain

Kim Prud'homme[a], Mary Alexandrou[b], Zsofia Grandpierre[c], and Julie Boucher[d]

[a]Kim Prud'homme, MSc OT, Children's Hospital of Eastern Ontario, Chronic Pain Service, Ottawa, Ontario, Canada; [b]Mary Alexandrou, M.Ed, Children's Hospital of Eastern Ontario, YouthNet, Ottawa, Ontario, Canada; [c]Zsofia Grandpierre, M.Ed, Children's Hospital of Eastern Ontario, YouthNet, Ottawa, Ontario, Canada; [d]Julie Boucher, MSc,Children's Hospital of Eastern,OntarioChronic Pain Service, Ottawa, Ontario, Canada

The Children's Hospital of Eastern Ontario (CHEO) Chronic Pain Team, in collaboration with YouthNet, an Ottawa-based mental health promotion and intervention program, endeavored to create a program that would address the specific needs of the chronic pain patient population. As such, a needs assessment focus group was held at YouthNet Ottawa to get input from the youth who utilize the service. Participants included past and current CHEO chronic pain patients between the ages of 11 and 18 ($n$ = 12). Gaps in the chronic pain program were discussed and a common theme that arose was the need for more opportunities to connect with peers.

From this, the Chronic Pain Team created a peer support group for youth experiencing chronic pain. Monthly drop-in sessions will allow for youth to meet and converse with their peers, to foster positive coping mechanisms. The sessions will be held at YouthNet in Ottawa starting in January of 2018 and be facilitated by YouthNet facilitators/front line youth workers as well as a young adult living with chronic pain. This peer group will be part of a pilot study which will evaluate participant satisfaction and program effectiveness. Preliminary survey data will be analyzed and presented herein, and will serve to further tailor the program as part of a larger, more comprehensive study of the effectiveness of a peer group on pain management and coping skills in a pediatric population.

**CONTACT** Kim Prud'homme kprudhomme@cheo.on.ca

© 2018 Kim Prud'homme, Mary Alexandrou, Zsofia Grandpierre and Julie Boucher. Published with license by Taylor & Francis Group, LLC.
This is an Open Access article distributed under the terms of the Creative Commons Attribution License (http://creativecommons.org/licenses/by/4.0/), which permits unrestricted use, distribution, and reproduction in any medium, provided the original work is properly cited.

### Learning through safety stories: Incorporating principles of safe opioid practice into PCA education for nurses

Jacqueline Hanley[a] and Sara McEwan[b]

[a]Department of Anaesthesia and Pain Medicine, SickKids, Toronto, ON, Canada; [b]Learning Institute, SickKids, Toronto, ON, Canada

Safe, effective pain management is a priority issue at SickKids, with recent attention paid to safe opioid practices. Aligning with SickKids' journey to eliminate preventable harm, the aim of this quality improvement initiative was to promote a culture of safety within pain- and opioid-related education. As many opioid-related errors that occur at SickKids involve PCA (Patient-Controlled Analgesia) therapy, the PCA class provided an ideal opportunity to address this aim. The class is a small group, 1.5 hour session offered multiple times per year to new and existing nursing staff. Historically, the class combined lectures with some hands-on activities, focusing on practical and procedural knowledge.

The class was redesigned as a case-based, interactive session, situating course content within real clinical contexts. Real safety events are used to encourage critical thinking, teach dose and weight-based calculations, discuss strategies for error prevention, and emphasize to learners the relevance of the course content.

Outcomes include nurses' engagement and satisfaction with the course content, nurses' confidence in being able to safely program PCA pumps and monitor patients, and awareness of common PCA-related errors and the knowledge to prevent them. To date, evaluations suggest high participant satisfaction and reflect positively on the inclusion of error prevention strategies, safety stories, hands-on learning and practice calculations. Many participants have expressed a desire for additional calculation-based cases to practice independently.

Next steps include the development of an independent learning package that includes additional case-based calculations. PCA-related errors will continue to be monitored across the hospital.

**CONTACT** Jacqueline Hanley jacqueline.hanley@sickkids.ca

© 2018 Jacqueline Hanley and Sara McEwan. Published with license by Taylor & Francis Group, LLC.
This is an Open Access article distributed under the terms of the Creative Commons Attribution License (http://creativecommons.org/licenses/by/4.0/), which permits unrestricted use, distribution, and reproduction in any medium, provided the original work is properly cited.

### Paediatric Project ECHO: assessing the educational needs of healthcare providers related to management of paediatric pain

Chitra Lalloo[a,c], Fiona Campbell[b], Jennifer Tyrrell[b], Annie Jiwan[a], Adam McKillop[a], Erinn McCarthy[a], and Jennifer Stinson[a]

[a]Child Health Evaluative Sciences, The Hospital for Sick Children, Toronto, Canada; [b]Anesthesia and Pain Medicine, The Hospital for Sick Children, Toronto, Canada; [c]Institute for Health Policy Management and Evaluation, The University of Toronto, Toronto, Ontario

**Introduction/Aim**: Project ECHO® is an innovative model for medical education that expands access and capacity to provide evidence-informed care. This model aims to increase knowledge, build capacity and, ultimately, improve patient outcomes by bringing expertise to the community. This project focuses on the first paediatric implementation of Project ECHO focused on management of acute and chronic pain. In preparation for program launch (October 2017), a needs assessment was conducted to: (1) inform the pain-specific ECHO curriculum; and (2) assess community healthcare provider (HCP) preferences for program format.

**Methods**: An online survey (49 items; 15-minutes) was distributed via targeted emails to established pain programs as well as professional networks, associations, and allied-health organizations related to pain throughout Ontario between May and August of 2017.

**Results**: N = 35 HCPs completed the survey. Profession breakdown was: 40% allied-health, 34% nurses, and 26% physicians. Overall, 43% of participants practiced in academic hospitals, 22% in non-academic hospitals, and 35% in other settings. Topics of interest included: musculoskeletal pain, mindfulness, mind-body techniques, and cognitive behavioural therapy. In terms of program format, 41% preferred weekly 1-hour educational sessions versus longer sessions.

**Discussion/Conclusions**: Community HCPs who expressed interest in *Paediatric Project ECHO for Children with Pain* came from diverse professional backgrounds, and generally preferred short educational sessions. The themes that emerged from this needs assessment spanned the physical, psychological, and pharmacological domains of managing paediatric pain patients. Survey results were used to inform a robust paediatric pain curriculum that is being delivered to meet the needs of community HCPs.

**CONTACT**T Chitra Lalloo chitra.lalloo@sickkids.ca

© 2018 Chitra Lalloo, Fiona Campbell, Jennifer Tyrrell, Annie Jiwan, Adam McKillop, Erinn McCarthy and Jennifer Stinson. Published with license by Taylor & Francis Group, LLC.
This is an Open Access article distributed under the terms of the Creative Commons Attribution License (http://creativecommons.org/licenses/by/4.0/), which permits unrestricted use, distribution, and reproduction in any medium, provided the original work is properly cited.

## A case report of serotonin toxicity diagnosed by inpatient chronic pain consultation

Matthew Sheppard[a], Jane Burns[b], and David Flamer[c]

[a]Department of Anesthesia, University of Toronto, Toronto, Ontario, Canada; [b]Sinai Health Systems, Wasser Pain Management Center, Toronto, Ontario, Canada; [c]Department of Anesthesia, University of Toronto, Toronto, Ontario, Canada

Serotonin Toxicity is a potentially life-threatening and severe condition caused by excess of the neurotransmitter serotonin. The diagnosis remains clinical and is based on the triad of autonomic hyperactivity, neuromuscular sequelae and acute mental status changes. We describe a case of serotonin toxicity that was diagnosed by the chronic pain service of a tertiary care hospital. The patient who had a history of major depressive disorder and chronic lower back pain was admitted to hospital with lobar pneumonia. After an initial improvement with antibiotics, the patient deteriorated and developed acute mental status changes, tachycardia and spontaneous clonus. The chronic pain service was consulted as this presentation was thought to be a consequence of medications. While the most likely implicated medications (Fentanyl patch, duloxetine and escitalopram) had not had their doses changed recently, he was noted to be on high doses of these medications with decreased organ function. After a chronic pain consultation occurred, the clinicians recognized the diagnosis of serotonin toxicity and advice was provided to the attending team. After a decrease in the fentanyl patch and temporary cessation of his escitalopram and duloxetine, the patient recovered from his symptoms and was able to be safely discharged home. Many case reports have been presented demonstrating the link between opioids and serotonin toxicity, specifically fentanyl. This case demonstrates the importance of chronic pain providers to be familiar with the diagnosis and management of serotonin syndrome as it can be a frequent presentation for patients who are co-prescribed opioids and antidepressants.

**CONTACT** Matthew Sheppard msheppard@munmed.ca University of Toronto, Department of Anesthesia, Toronto, Ontario, Canada,

© 2018 Matthew Sheppard, Jane Burns and David Flamer. Published with license by Taylor & Francis Group, LLC.
This is an Open Access article distributed under the terms of the Creative Commons Attribution License (http://creativecommons.org/licenses/by/4.0/), which permits unrestricted use, distribution, and reproduction in any medium, provided the original work is properly cited.

## Changes in the opinion and potential use of medicinal cannabis amongst chronic pain patients after receiving education by a pharmacist

Vikas Parihar[a], Li Wang[b], Laura Katz[c], and Lisa Patterson[a]

[a]Department of Anesthesia, Michael G. DeGroote Pain Clinic, McMaster University, Hamilton, Ontario, Canada; [b]Department of Anesthesia, Michael G. DeGroote Institute of Pain Research and Care, McMaster University, Hamilton, Ontario, Canada; [c]Department of Psychology & Behavioural Neuroscience, Michael G. DeGroote Pain Clinic, McMaster University, Hamilton, Ontario, Canada

**Introduction**: To better inform patients and encourage safer use of medicinal cannabis, the Michael G. DeGroote (MGD) Pain Clinic provides a one hour Medical Cannabis Education Session (MCES) by a pharmacist before receiving a medical document.

**Methods**: Patients were surveyed pre and post a MCES between June-November 2017 as part of a quality improvement initiative. The McNemar's test was used to analyze the differences before and after the MCES.

**Results**: One hundred and twenty patients completed the survey, with a mean age of 53 ± 12.42 years, 58% were female, and 42% were currently using cannabis (either recreational or medicinal). After attending a MCES, more patients believed cannabis was possibly addictive (61.7% vs 35.8%, p < 0.001) and harmful (50% vs 31.6%, p = 0.001), but potentially better than narcotics for their chronic pain condition (41.4% vs 24.3%, p < 0.001). No significant change in the opinion of medicinal cannabis and its comparative safety to narcotics was noted (p = 0.27). After the MCES, more patients indicated they would use a low-medium concentration of THC (55.6% vs 20%, p < 0.001) and use a medium-high concentration of CBD (77.3% vs 27.3%, p = 0.003) than prior to the MCES. Eighty percent of patients found the MCES helpful.

**Conclusions**: After the MCES, significantly more participants would use a low-medium THC/medium-high CBD product. In addition, patients were more likely to report that cannabis would be better than narcotics for pain control, but could also be addictive and harmful. Overall, education by a pharmacist was able to establish safer perceptions and potential use of medicinal cannabis.

**CONTACT** Vikas Parihar parihav@mcmaster.ca Department of Anesthesia, Michael G. DeGroote Pain Clinic, McMaster University, Hamilton, Ontario, Canada

© 2018 Vikas Parihar, Li Wang, Laura Katz and Lisa Patterson. Published with license by Taylor & Francis Group, LLC.
This is an Open Access article distributed under the terms of the Creative Commons Attribution License (http://creativecommons.org/licenses/by/4.0/), which permits unrestricted use, distribution, and reproduction in any medium, provided the original work is properly cited.

### Interventional cancer pain, not the 4th step but the handrail to the WHO analgesic ladder

Uri Hochberg[a], Yoram Shir[b], Louis M. Boucher[a], and Jordi Perez[a]

[a]Cancer Pain Clinic, McGill University Health Centre, Montreal, Quebec, Canada; [b]Alan Edwards Pain Management Unit, McGill University Health Centre, Montreal, Quebec, Canada

**Introduction**: Interventional cancer pain procedures are offered to roughly 1/3 of patients treated at McGill University Cancer Pain Clinic. We offer this approach at earlier stages rather than waiting for many drugs to fail or before the patient is too ill. We present a retrospective analysis of procedures offered to cancer pain patients during 21 consecutive months.

**Methods**: chart review of cases seen in clinic between June 2015 and March 2017 and scheduled for a block. Demographic data, cancer status, symptoms ratings (using ESAS) and opioid consumption were analyzed before and after blocks and between responders and non-responders.

**Results**: 127 procedures were reviewed and 82 were included in this analysis. Most patients had metastatic gastrointestinal cancer (76%). Eighty percent of patients reported an improvement in their pain and 43% were considered responders (≥50% relief).

Compared to the non-responders, responders reported an average 4.6/10 points decreased pain (68%), improvement in fatigue, depression, anxiety and drowsiness, and enhanced sensation of wellbeing. Finally, half of the responders stopped using short acting PRN opioids.

**Conclusions**: interventional cancer pain approaches are valuable and effective analgesic options that should not be restricted solely to drug-resistant cases. Rather than a fourth step, these approaches should be regarded as the handrail to the WHO cancer pain ladder.

**CONTACT** Uri Hochberg urihocberg@hotmail.com

© 2018 Uri Hochberg, Yoram Shir, Louis M. Boucher and Jordi Perez. Published with license by Taylor & Francis Group, LLC.
This is an Open Access article distributed under the terms of the Creative Commons Attribution License (http://creativecommons.org/licenses/by/4.0/), which permits unrestricted use, distribution, and reproduction in any medium, provided the original work is properly cited.

### Opioid risks & benefits - A video and slide deck

John X. Pereira[a], Joyce Cote[a], Michelle M. DeLisle[a], Deon Louw[b], Robert Tanguay[b], and Yoram Shir[c]

[a]Calgary Chronic Pain Centre, Calgary, Alberta, Canada; [b]Caleo Health, Calgary Alberta; [c]The Alan Edwards Pain Management Unit, Montreal, Quebec

Created with the input and support of over thirty healthcare providers from three countries, Opioid Risks & Benefits - A Guide for Patients with Chronic Pain is a new resource. Comprising of a slide deck for healthcare professionals as well as a complete video for patient viewing, this endeavor provides patients with an up-to-date, balanced, evidence-informed presentation on the use of opioids for chronic pain. Addressing issues such as tolerance, hormone effects, overdose, mood change risks and falls, this presentation has been well-accepted by patients and providers and nominated for an Alberta Health Services President's Excellence Award. Patient video accessible at opioidinfo.ca.

**CONTACT** John X. Pereira john@johncpc.com Calgary Chronic Pain Centre, Calgary, Alberta, Canada

© 2018 John X. Pereira, Joyce Cote, Michelle M. DeLisle, Deon Louw, Robert Tanguay, and Yoram Shir. Published with license by Taylor & Francis Group, LLC.
This is an Open Access article distributed under the terms of the Creative Commons Attribution License (http://creativecommons.org/licenses/by/4.0/), which permits unrestricted use, distribution, and reproduction in any medium, provided the original work is properly cited.

### FibroFOCUS: an interdisciplinary fibromyalgia treatment program

John X. Pereira, Diane Marie Colley-Urquhart, Joyce Cote, Arlene Cox, W. Jeptha Davenport, Adrian Gretton, Kara Hallett, Magali Robert, and Diane Roylance

Calgary Chronic Pain Centre, Alberta Health Services, Calgary, Alberta, Canada

**Background**: Fibromyalgia is a chronic pain condition affecting an estimated 3.3% of the adult Canadian population. It is considered challenging to treat and interdisciplinary care is mentioned in the Canadian guidelines, but outcome data relating to such approaches are scarce.

**Methods**: From Aug 2014 to Dec 2016, a total of 50 individuals with fibromyalgia were enrolled in a 13-week treatment program involving interdisciplinary care from a nurse, physical therapist, kinesiologist, occupational therapist, pharmacist, dietitian, psychologist and physician. Patients received approximately 100 hours of treatment from the interdisciplinary team, mostly as part of once-weekly groups with limited one-on-one care.

**Results**: Short-term outcome data was available on 45 individuals. Compared to pre-treatment, immediate post-treatment Fibromyalgia Impact Questionnaire (FIQ-R) scores were significantly decreased, from 67.5 to 52.1 ($N = 45$, $p < 0.001$). Depression scores on Patient Health Questionnaire (PHQ-9) were reduced from 15.6 to 12.2 ($N = 44$, $p < 0.001$). Pain self-efficacy (PSEQ) increased from 28.8 to 34.0 ($N = 43$, $p < 0.001$).

**Conclusions**: Our data show that fibromyalgia is amenable to group-based interdisciplinary treatment with clinically significant short-term improvement in FIQ-R, mood, sleep and self-efficacy. There is a need for rigorous measurement of long-term outcomes of this approach.

**CONTACT** Diane Marie Colley-Urquhart Colley-Urquhart@ahs.ca RN Clinical Nurse Team Leader Diane.

© 2018 John X. Pereira, Diane Marie Colley-Urquhart and Kara Hallett. Published with license by Taylor & Francis Group, LLC.
This is an Open Access article distributed under the terms of the Creative Commons Attribution License (http://creativecommons.org/licenses/by/4.0/), which permits unrestricted use, distribution, and reproduction in any medium, provided the original work is properly cited.

## Road to recovery: empowering and educating staff and patients by positioning pain management as foundational to recovery

Jill Campbell[a], Donna Bouchard[b], Heather Russek[c], Cheryl Hsu[d], Laura Tomat[e], Lourdes Rodrigo[b], Dorothy Labak[b], and Alies Maybee[f]

[a]Acute Pain Service, St. Joseph's Health Centre, Toronto, Canada; [b]Surgical Program, St. Joseph's Health Centre, Toronto, Canada; [c]Corporate Strategy and Strategic Initiatives, St. Joseph's Health Centre, Toronto, Canada; [d]Strategic Foresight and Innovation, Design Researcher and Strategist, Toronto, Canada; [e]Research, St. Joseph's Health Centre, Toronto, Canada; [f]Patient and Family Advisor, St. Joseph's Health Centre, Toronto, Canada

The *Road to Recovery* initiative began at St. Joseph's Health Centre Toronto when an opportunity was recognized to empower and educate surgical patients and their families around improving their journey to recovery and preparedness for discharge. Co-led by a team of Advanced Practice Nurses, Administrator, Researcher, Patient-family advisors, and a Designer, *Road to Recovery* was developed.

The initiative began with a problem discovery phase which involved interviews with 11 staff and 14 patient families as well as observations in pre-admission, day of surgery, family waiting area, recovery room, and the surgical unit. This enabled an understanding of the experience of the surgical patients' journey, and led to the insight that pain management has to be addressed as a priority during recovery in order for other recovery goals to be met. A recovery hierarchy positioning pain management as foundational to successful recovery was developed as a communication tool for patients, family, and staff.

*Road to Recovery* empowers patients to take ownership of their recovery and facilitates interdepartmental staff communication around pain. A patient journal and posters for patients, families, and staff were created to reinforce learning. A pilot program is underway to evaluate the following objectives: 1) To determine if the *Road to Recovery* initiative empowers and educates patients on their recovery and preparedness for discharge; 2) To determine if by educating patients about their medications and associated side effects, they can take a collaborative role in their recovery; 3) To assess patient, family, and interdepartmental staff uptake of the initiative.

**CONTACT** Jill Campbell JCampbell2@stjoestoronto.ca

© 2018 Jill Campbell, Donna Bouchard, Heather Russek, Cheryl Hsu, Laura Tomat, Lourdes Rodrigo, Dorothy Labak and Alies Maybee. Published with license by Taylor & Francis Group, LLC.
This is an Open Access article distributed under the terms of the Creative Commons Attribution License (http://creativecommons.org/licenses/by/4.0/), which permits unrestricted use, distribution, and reproduction in any medium, provided the original work is properly cited.

## Improving efficiency of consultations in a chronic pain setting: the role of the physician assistant

Jaclyn Millar

Canadian Certified Physician Assistant, Bachelor of Science Physician Assistant, Honours Bachelor of Science Kinesiology, McMaster University Medical Centre, Michael G. Degroote Pain Clinic, Hamilton, ON, Canada

The Physician Assistant (PA) profession is currently being incorporated into the Canadian health care system. Responsibilities and duties vary depending on the medical setting, the scope of practice of their supervising physician, and the laws of the province in which they practice. PA's help address substantial challenges facing patients seeking timely access to quality health care services. The collaborative approach between the Physician and the PA reduces the workload placed on the physician and allows them to focus their efforts where needed while the PA can provide support in other needed areas. In 2014–15, the wait time to see a pain physician was over 2 years. The number of new consult and follow up visits have increased from 2,560 in 2014 to 7,040 in 2016–17 (over a 12 month period), which represents a 2.75 fold increase, since the addition of the PA. The PA works collaboratively with 10 physicians on an alternating schedule and sees an average of 70% of all new consultations and 22% of all follow up appointments, reducing the overall wait time to an average of 16 weeks for a new consult. In addition to this dramatic reduction in new consult wait time, the pain physician is now able to see follow up patients much sooner. Overall, the data supports the value and role of the PA within the MGD Pain clinic, as this allows a patient to be seen in a timely fashion and also addresses the issue of access to specialized chronic pain care treatment.

**CONTACT** Jaclyn Millar millarjac@hhsc.ca Honours Bachelor of Science Kinesiology, McMaster University Medical Centre, Michael G. Degroote Pain Clinic, Hamilton, ON, Canada.

© 2018 Jaclyn Millar. Published with license by Taylor & Francis Group, LLC.
This is an Open Access article distributed under the terms of the Creative Commons Attribution License (http://creativecommons.org/licenses/by/4.0/), which permits unrestricted use, distribution, and reproduction in any medium, provided the original work is properly cited.

## Evaluation of a clinical pathway using intranasal fentanyl for treatment of vaso-occlusive crisis in sickle cell patients in the emergency department

Hugo Paquin[a], Evelyne D Trottier[a], Yves Pastore[b], Nancy Robitaille[b], Marie-Joelle Dore Bergeron[c], and Benoit Bailey[a]

[a]Department of Pediatric Emergency Medicine, CHU Ste-Justine, Montréal, Canada; [b]Department of Hematology and Oncology, CHU Ste-Justine, Montréal, Canada; [c]Department of Pediatrics, CHU Ste-Justine, Montréal, Canada

**Background**: Sickle cell vaso-occlusive crisis (VOC) is a frequent cause of emergency visit in children. Intranasal (IN) fentanyl has been used increasingly for pain treatment in the emergency department (ED).

**Objectives**: We aim to evaluate whether the use of a new innovative pain management pathway using IN fentanyl from triage for patients with sickle cell disease (SCD) in VOC lead to improved care.

**Methods**: Retrospective chart review of patients with SCD who presented to the ED with VOC, in the period pre and post implementation of the protocol.

**Results**: A total of 96 ED patients were included (52 pre, 44 post). Using IN Fentanyl, patients received a first dose of opiate within 52.3 minutes of arrival (IQR 30.6, 74.6), corresponding to a 41.4 min reduction in the opiate administration time (95% CI –56.1, –27.9). There was also a 43% increase in the number of patient treated with a non-IV opiate as 1st opiate dose (95% CI 26, 57). In patients who were discharged from the ED, there was a 49% decrease in the number of IV-line insertion (95% CI −67, −22). There was no difference in the hospitalization rates (difference of 6 (95% CI −13, 25)).

**Conclusions**: This study validates the use of our protocol using IN fentanyl for treatment of VOC in the ED by significantly reducing the time to 1st opiate dose, now meeting quality of care indicators. Our standardized protocol using IN Fentanyl is a safe approach. Still, its safety should be further evaluated in prospective studies.

### ORCID

Hugo Paquin http://orcid.org/0000-0001-9877-903X

**CONTACT** Hugo Paquin hpaquin@icloud.com

© 2018 Hugo Paquin, Evelyne D Trottier, Yves Pastore, Nancy Robitaille, Marie-Joelle Dore Bergeron, and Benoit Bailey. Published with license by Taylor & Francis Group, LLC.
This is an Open Access article distributed under the terms of the Creative Commons Attribution License (http://creativecommons.org/licenses/by/4.0/), which permits unrestricted use, distribution, and reproduction in any medium, provided the original work is properly cited.

## Optimizing patient flow from referral to initial assessment at an academic hospital-based interdisciplinary chronic pain clinic

Kyle Vader, Elizabeth Brown, Emily Curtis, Jessica Allison, Etienne J. Bisson, Tom Doulas, Mary Anne Good, Rebecca McDermott, Kerry Oulton, Colleen Saunders-Cartier, Christine Varey, and Scott Duggan

Kingston Health Sciences Centre-Hotel Dieu Hospital site, Chronic Pain Clinic, Kingston, Ontario, Canada

**Background**: In an effort to direct the right patient, at the right time, to the right healthcare providers, the Chronic Pain Clinic at the Hotel Dieu Hospital site of Kingston Health Sciences Centre in Kingston, Ontario is piloting a new intake process, which includes a new referral form, a phased triage system, and a new patient orientation session.

**Referral Form**: A revamped referral form that asks the referrer to provide a more thorough medical history, social history, and summary of objective findings and tests is being introduced with the hope of obtaining a more robust referral.

**Triage Phase I**: A registered nurse evaluates the appropriateness, complexity, and urgency of each new referral.

**Orientation Session**: Two healthcare professionals (e.g. a physiotherapist and registered nurse) deliver a standardized 120-minute didactic presentation to new patients. The goal of the orientation session is to (1) highlight the importance of an active versus passive pain management strategies, (2) establish realistic expectations for pain management, and (3) introduce key concepts of pain neuroscience education. New patients also complete self-reported outcome measures at this visit.

**Triage Phase II**: After a new patient attends an orientation session, a detailed file review is conducted by a registered nurse. The patient is then offered an initial assessment with either a single healthcare provider or an interdisciplinary team as determined by the file review.

**Future steps**: Future steps include conducting a formal evaluation to determine the impact of this new patient flow on the patient experience, wait-times, and healthcare service usage.

**CONTACT** Kyle Vader kyle.vader@kingstonHSC.ca Kingston Health Sciences Centre-Hotel Dieu Hospital site, Chronic Pain Clinic, Kingston, Ontario, Canada

© 2018 Kyle Vader, Elizabeth Brown, Emily Curtis, Jessica Allison, Etienne J. Bisson, Tom Doulas, Mary Anne Good, Rebecca McDermott, Kerry Oulton, Colleen Saunders-Cartier, Christine Varey and Scott Duggan. Published with license by Taylor & Francis Group, LLC.
This is an Open Access article distributed under the terms of the Creative Commons Attribution License (http://creativecommons.org/licenses/by/4.0/), which permits unrestricted use, distribution, and reproduction in any medium, provided the original work is properly cited.

## Predictors of readiness for change in an interdisciplinary chronic pain program

Laura Katz and Lisa Patterson

Michael G. DeGroote Pain Clinic, McMaster University Medical Centre, Hamilton, ON, Canada

**Introduction/Aim**: Interdisciplinary chronic pain management is considered to be the gold standard of treatment. The Michael G. DeGroote (MDG) Pain Clinic offers an interdisciplinary pain program for individuals with chronic pain. Participant's readiness for change impacts their ability to engage and benefit from the Program. The aim of this study was to evaluate predictors of the stages of change to determine potential targets to increase engagement in the Program.

**Methods**: Questionnaires were administered and completed by patients attending their first day of an 8-day Interdisciplinary Chronic Pain Program. Data were analyzed using descriptive statistics and multiple linear regressions.

**Results**: Ninety-five percent (76/80) of participants completed questionnaire packages. Pre-contemplation ($F = 3.05$, $p < 0.01$) was significantly predicted by fear of pain/re-injury ($\beta = 0.31$, $p = 0.02$), and not pain intensity, depression, anxiety, stress, coping, or catastrophizing. The action stage of change ($F = 3.96$, $p < 0.01$) was significantly predicted by depression ($\beta = 2.88$, $p < 0.01$), fear of pain/re-injury ($\beta = 2.41$, $p = 0.02$), and catastrophizing ($\beta = 2.82$, $p < 0.01$), and not intensity of pain, anxiety, stress, or coping.

**Discussion/Conclusions**: Finding predictors related to the beliefs that pain is a medical problem and should be treated by physicians versus those who are engaged and ready to take action in a self-management approach helps to identify targets for intervention. Future research should examine the benefits of pre-intervention treatment of fear of pain/re-injury, depression and catastrophizing on post-program outcomes.

**CONTACT** Laura Katz katzl@hhsc.ca Michael G. DeGroote Pain Clinic, McMaster University Medical Centre, Hamilton, ON, Canada.

© 2018 Laura Katz and Lisa Patterson. Published with license by Taylor & Francis Group, LLC.
This is an Open Access article distributed under the terms of the Creative Commons Attribution License (http://creativecommons.org/licenses/by/4.0/), which permits unrestricted use, distribution, and reproduction in any medium, provided the original work is properly cited.

## The development of a buprenorphine/naloxone induction clinic in a tertiary pain clinic

Joyce Lee, Naomi Steenhof, Nivan Zoheiry, and John Flannery

Comprehensive Integrated Pain Program, Toronto Rehabilitation Institute, Toronto, Ontario, Canada

**Introduction/Aim**: This poster examines the effectiveness and safety of prescribing buprenorphine/naloxone for patients with chronic pain at an interdisciplinary pain clinic.

**Methods**: A buprenorphine/naloxone induction clinic was developed by a nurse practitioner, physiatrist and pharmacist within an existing pain clinic. All patients met the DSM-5 diagnostic criteria for opioid use disorder and had complex medical histories including coexisting psychiatric diagnoses and/or sleep disorders. Eligible patients included individuals with chronic pain who were taking <200mg morphine equivalents daily, were experiencing tolerance and/or side effects to their prescribed opioids and were unsuccessful in decreasing their opioid intake after at least one weaning attempt. Patients were seen for a total of six visits for education, induction, rapid titration and follow-up. Severity of pain and its impact on functioning were monitored by the brief pain inventory (BPI), withdrawal was assessed using the clinical opiate withdrawal scale (COWS), and adherence was assessed by urine drug screen.

**Results**: The majority of patients experienced an improvement in functional scores as assessed by the BPI, fewer side-effects, and decreased risk of overdose. Common side effects from the buprenorphine/naloxone induction included headaches and mild dizziness.

**Discussion/Conclusion**: A buprenorphine/naloxone clinic tailored to meet the needs of individuals with chronic pain can be successful in stabilizing verbal pain scores and increasing functional scores. This clinic demonstrated an improved safety profile for patients and minimized withdrawal symptoms; however, it was resource intensive from an administrative and clinical perspective. Exploration into improving patient flow is ongoing.

**CONTACT** Joyce Lee joyce.lee@uhn.ca Comprehensive Integrated Pain Program, Toronto Rehabilitation Institute, Toronto, Ontario, Canada.

© 2018 Joyce Lee, Naomi Steenhof, Nivan Zoheiry and John Flannery. Published with license by Taylor & Francis Group, LLC.
This is an Open Access article distributed under the terms of the Creative Commons Attribution License (http://creativecommons.org/licenses/by/4.0/), which permits unrestricted use, distribution, and reproduction in any medium, provided the original work is properly cited.
